# Greenpeace and the online genetically modified food debate in the UK: The role of science and scientific evidence in ‘environmental representation’

**DOI:** 10.1177/09636625221138765

**Published:** 2022-12-15

**Authors:** Catherine Price

**Affiliations:** University of Nottingham, UK

**Keywords:** claims-making, environmental representation, GM food, Greenpeace, news media, scientific evidence

## Abstract

This article investigates Greenpeace’s use of science in ‘environmental representation’ in news articles concerning genetically modified food, and how commenters respond in the associated below the line comments. This article provides an answer through a qualitative data analysis using a discourse analysis of data from 5 UK news organisations, commencing 1 January 2015 until 31 December 2015. The findings reveal the importance of science and scientific evidence in claims-making and ‘environmental representation’ by Greenpeace in relation to genetically modified foods in news articles. However, below the line commenters reject the idea of scientific evidence being used by Greenpeace. Instead, these commenters claim Greenpeace oppose scientific developments. The article concludes by discussing how this study adds to the understanding of claims-making and ‘environmental representation’ by Greenpeace with respect to genetically modified foods in news articles, and how the below the line commenters challenge the legitimacy of Greenpeace as an ‘environmental representative’.

## 1. Introduction

The environmental movement is concerned with the use of the Earth’s resources and the exploitation of the planet. As well as environmental non-governmental organisations (NGOs), some humanitarian NGOs fall under this umbrella where livelihoods are trying to be protected. Reflecting on the scientific aspects of the genetically modified (GM) food debate, it is worth considering how scientific evidence is evaluated by NGOs and social movements. Durant (1998) argues that a ‘sceptical attitude to science is all around us. It is apparent, for example, in the increasing confidence with which pressure groups such as Friends of the Earth and Greenpeace contest scientific evidence on environmental issues’ (p. 72).

Studies have been conducted concerning claims-making and framing of Greenpeace and environmental issues. These include the ‘Brent Spar’ controversy (Hansen, 2000), and the golden rice experiment in China (Yang et al., 2014). While these studies have analysed news articles, there is a lack of research concerning news articles and the associated below the line comments. There is also a lack of research concerning expertise and NGOs (Eden et al., 2006). The aim of this article is to investigate Greenpeace’s use of science in environmental representation in news articles concerning GM food, and how commenters respond in the associated below the line comments.

Section two discusses the literature concerning claims-making and environmental representation, and digital news and below the line comments. The third section discusses the methods and the research design, while the findings of the research and discussion are outlined in section four. In the final section, I draw conclusions as to how this study adds to our understanding of claims-making and environmental representation by Greenpeace with respect to GM foods in news articles, and how the below the line commenters challenge the legitimacy of Greenpeace as an environmental representative.

## 2. Literature review

### Claims-making and environmental representation

Issues which make it onto a news organisation’s agenda often do so because these matters are considered problematic. Many journalists like reporting conflicts because they provide the opportunity for making the powerful uncomfortable (Schudson, 2008). In terms of conflicts, Spector and Kitsuse (1973) define social problems as ‘*the activities of groups making assertions of grievances and claims to organisations, agencies, and institutions about some putative conditions*’ (p. 146, emphasis in original). The emergence of a social problem depends on defining a putative situation or condition as a problem, and removing, altering or improving that situation or condition. As Hannigan (2014) argues, from this perspective ‘the process of claims-making is treated as more important than the task of assessing whether these claims are truly valid or not’ (p. 51). Claims are ‘complaints about social conditions that members of a group perceive to be offensive and undesirable’, and this definition arises from Spector and Kitsuse’s original concept (Hannigan, 2014: 51). Claims-makers are those who make claims and these include victims, activists, specialists, professionals, pressure groups, and officials (Best, 2002). Different claims-makers can forge alliances to promote a particular problem (Hannigan, 2014). Claims-makers convey their claims using *language* they believe is persuasive and which they consider their audience to find persuasive. When there is a debate about a particular social problem, rival claims-makers will offer different characterisations about the debate, making the audience decide who to believe (Best, 2002). Claims-making relates to the different activities claims-makers undertake in order to bring claims to the attention of the audience (Hannigan, 2014).

The environment or nature cannot make claims, but instead has to rely on representation. For Tanasescu (2014), representation is ‘primarily – structurally – about relations. These are connected to the logic of claim-making and rely on the power of linguistic proclamation’ (p. 40). Through the processes of claims-making and the acceptance or rejection of claims by audience members, representation is produced (Saward, 2006). It is important to recognise that claims are contestable and can be contested, and so too, can representation. Here, it is useful to consider Michael Saward’s explanation of representative claims-making. When making a representative claim, a ‘**maker** of representations **(M)** puts forward a **subject (S)** which stands for an **object (O)** which is related to a **referent (R)** and is offered to an **audience (A)**’ (Saward, 2006: 302, emphasis in original). The person making the representative claim will wish to see it accepted, but the audience is always free to interpret it as they see fit. Here, the audience uses rules of recognition^1^ to decide whether the person making the claim is a representative in that certain case (Boström and Uggla, 2016; Rehfeld, 2006).

Actors making claims about the environment or nature act as representatives (Boström and Uggla, 2016). Environmental or nature representation is not straightforward and is faced with practical challenges such as who speaks for future generations and the more-than-human world (Boström and Uggla, 2016; Carolan, 2006; O’Neill, 2001). This raises the question, who can legitimately claim to speak on behalf of the environment or nature? (O’Neill, 2001). Here, it is important to acknowledge that any person or organisation can claim to be representing the environment or nature (Boström et al., 2018). Representatives may speak officially on behalf of organisations, or may do so informally through affiliations. Representatives may also act symbolically as a role model (Boström and Uggla, 2016).

Multiple identities are present in representative claims-making. The process is also dynamic when representatives engage and debate an issue. However, as Boström and Uggla (2016) argue, while ‘there can never be any complete, impartial and neutral representation, it would at the same time be dubious to say that all representations are equally useful, relevant, effective and democratic’ (p. 363). The significance of a claim depends not only on a convincing and powerful argument, but also who is making the claim (Alcoff, 1991). For example, a scientist making an environmental claim is likely to be considered more significant than an informed citizen. Who is making a claim determines the style and language used, so this can also impact the claims significance (Alcoff, 1991). Not only will style and language use play a role, but the presentation of a claim will depend on the objectives of the representative. Knowledge straddles beliefs about what exists and what we think ought to be (Carolan, 2004). Therefore, representatives may present claims about what they believe the situation should be as opposed to the current reality.

Environmental problems are multifaceted. This complexity is further amplified because environmental facts cannot speak for themselves. Environmental facts are tied up with the social because a representative has to speak on behalf of the environment (Carolan, 2004). It is scientists, policymakers, NGOs and concerned citizens who give the environment and nature a voice. However, marginalised voices including women, children, ethnic minorities, and indigenous communities are often unable to speak about the environment (Carolan, 2004; O’Neill, 2001). This means environmental debates with a multiplicity of knowledge claims and voices are lacking. Instead, biologists, ecologists, natural scientists and environmental groups will often speak for those who are voiceless. This is because those ‘who claim to speak on behalf of those without voice do so by appeal to their having knowledge of the objective interests of those groups, often combined with special care for them’ (O’Neill, 2001: 496). However, it is worth noting that those speaking for the voiceless are often viewed as having the authority and knowledge to speak about a particular subject (Alcoff, 1991).

Before moving on, it is worth considering expertise in greater detail. Expertise is concerned with specialisation in the field of knowledge and in the production of knowledge. Expert systems according to Giddens (1991: 19) depend on trust which ‘presumes a leap to commitment, a quality of ‘faith’ which is irreducible’ and furthermore, ‘trust brackets the limited technical knowledge which most people possess about coded information which routinely affects their lives’. Trust allows people to go about their daily lives, mainly because everyone is effectively a lay person in virtually all social activities. As experts all have their own specialised knowledge, the accumulation of this as a whole allows us to trust that society will function. Of course, there are times when trust in experts is withdrawn, and people draw on their own knowledge to make decisions.

Giddens (1991) argues that the knowledge which is associated with expertise is in effect available to everyone providing they have the energy, time and resources to attain it. However, the most anyone can ever hope to achieve is to be an expert in one small, particular area. This narrow focus of expertise though, also gives rise to the problem of unexpected and unintentional consequences. Solving these difficulties means developing expertise further and therefore, a cycle emerges. Expertise can be defined as a knowledge which is relied upon by others and can be recognised by education, experience, peer support and talent. Although each one indicates expertise, it is the combination of them which provides an indication on who can be trusted (Nichols, 2017).

While experts and expertise are valued by society, it is equally possible that expert knowledge can be rejected. Turner (2001) argues because expertise is justified by legitimisation, it is also possible for this to work in reverse, and for the legitimacy of expertise to be retracted. The opinions of those considered to be experts can be disregarded.

It is also important to acknowledge that if the voiceless are relying on those who have the power and authority to speak, then there is the possibly that those people or objects being spoken for are further disempowered (Alcoff, 1991). Activities are required that attempt to address the lack of representation through the inclusion of a more diverse range of voices in debates (Carolan, 2006; O’Neill, 2001). This could potentially give the voiceless a voice.

### Digital news and below the line comments

Environmental news follows the same media routines and constraints as news in general (Berglez, 2011; Boykoff and Boykoff, 2004, 2007; Cox and Pezzullo, 2016; Hansen, 2010). In respect of how environmental journalism operates, Declan Fahy (2018) describes three points. First, since the 1960s, environmental journalists have tended to favour advocacy journalism as opposed to objective journalism. Advocacy journalism presents the news from a particular point of view, is motivated by a social or political agenda, and does not separate fact from values. Second, environmental journalism has addressed the issue of balance. Many environmental journalists no longer apply balance to their news stories, instead favouring the use of weight-of-evidence reporting. Weight-of-evidence reporting requires journalists to provide the audience with contrasting points of view and to report these accurately. However, the environmental journalist also has to determine the majority consensus in respect of evidence and report that to audiences (Dunwoody, 2005). A weight-of evidence narrative provides the audience with a range of claims about an issue, while making clear how experts view each of these claims (Dunwoody and Kohl, 2017). However, the effectiveness of this type of reporting relies on the audience trusting expert claims. Fahy’s (2018) third point is that environmental journalists have to decide on how to report controversial issues. Scientists who examine controversial issues from different disciplines, all produce valid scientific facts. Therefore, scientific evidence can be used to support a variety of positions on a problematic issue. In deciding on how to report a news story, environmental journalists face contradictory ideals, demands and expectations (Berglez, 2011).

Although the boundaries of professional journalism have been relatively stable, the different forms of obtaining online news have now made these more porous (Jukes, 2018). Digital news consumption enables those with access to devices such as computers, tablets and smartphones to access news whenever it is convenient. By its very nature, digital journalism requires a level of interaction, and there is a two-way relationship between producers and consumers. This is alluded to by Ksiazek and Peer (2017) who perceive digital journalism as a combination of traditional journalism with user-generated content and user-user interactions. Following the shift to digital platforms, scientists, NGOs, activists, and citizens can now actively contribute to news. This means ‘online science news and content has the potential to be highly participatory, social, and collaborative’ (Fahy and Nisbet, 2011: 782).

Many mainstream news media organisations in western democracies have adopted participatory forms of online journalism (Graham and Wright, 2015). News organisations which provide online news also often provide the facility of below the line comments. ‘Below the line’ is ‘industry parlance for the comment, and debate spaces opened up underneath news articles and blogs’ (Graham and Wright, 2015: 319). Below the line comments enable participatory journalism and can improve citizen participation and involvement in news making activities. Canter (2013) observes that ‘comment threads in particular have grown exponentially in recent years as readers have embraced the opportunity to bypass the Letters’ Editor and publish their opinions directly to a newspaper website’ (p. 604).

Below the line comments provide a means of allowing the audience to participate in a discussion about current events as well as offering competing headlines and interpretations to the news article (Ksiazek, 2018). Each comment is ‘anchored in somebody’s present in the sense that it signifies a more or less immediate reaction to the reading of an article and/or preceding comments to this article’ (Bødker, 2017: 60). Therefore, comments provide an opportunity for engagement and self-expression. However, these views can often be opposite to those which appear as the official consensus provided by experts in news articles (Turner, 2013). Responsible journalists explain expert knowledge in terms which the news reading audience can understand. By doing so, expert knowledge is made accessible, and legitimate claims are distinguished from those which are false. In contrast, the comments section ‘is open to anyone, has no filters, and allows false and misleading attacks on experts and assertions of fact that conflict with expert knowledge’ (Turner, 2013: 162).

However, this challenge to expert knowledge can potentially be a form of quiet activism (Price, 2021). Activism can take place in a setting such as the below the line comments when everyday aspects of life are considered forms of engagement and participation (Marres, 2015). Marres (2015) explains that participation occurs when ‘everyday things, devices and environments . . . *acquire* the capacity to engage and to mediate involvement with public affairs’ (p. 2, emphasis in original). Clicking and inserting a comment enables audience members to engage with a debate and for participation to occur (Marres, 2017).

## 3. Methods

A note on the methods: this article is based on a broader research project (Price, 2018). The methods described here are for the research project as a whole and this article forms one part of the findings. The research project ran from September 2014 to September 2018.

While the research was being undertaken, it became evident that GM crops and foods were providing a backdrop to a dispute between Professor Anne Glover and Greenpeace. Professor Glover was appointed to the role of European Union (EU) Chief Scientific Adviser in December 2011 and held the position until it was abolished in 2014. During 2015, the role of scientific advice for the European Commission was renegotiated and the Scientific Advice Mechanism was established. In 2016, the first seven Chief Scientific Advisers were appointed along with the establishment of Science Advice for Policy by European Academies (SAPEA). In retrospect, the changes to scientific advice for the European Commission during this time period, and specifically 2015, turned out to be fruitful for my research goals. The abolishment of the position of European Union Chief Scientific Adviser is the background to the newspaper coverage featured in the Findings and Discussion section. Major aspects of Professor Glover’s role included providing independent advice to the EU President on science, technology and innovation, and advising on novel science and technology, especially in terms of opportunities or threats to the EU. In order to address the aim of this article, which was to investigate Greenpeace’s use of science in environmental representation in news articles concerning GM food, and how commenters respond in the associated below the line comments, the following question was asked.

*Research Question*: How does Greenpeace use science in claims-making and environmental representation in news articles concerning GM food, and how do commenters respond in the associated below the line comments?

### Data collection and selection

A qualitative study was undertaken which included online news articles and the associated below the line comments from UK online news organisations. The news organisations included in the sample were *The Guardian; The Telegraph; The Times; The Daily Mail*; and *The Mirror*. These also included the Sunday editions. The sample included what have traditionally been seen as the broadsheets (*The Guardian, The Telegraph*, and *The Times*), and the tabloids (*The Daily Mail* and *The Mirror*). The news organisations included in the sample were chosen because of their diversity of content. The broadsheets generally are assumed to provide more in depth content, while the tabloids tend to be more concise and simplistic. The sampling time frame ran from 1 January 2015 until 31 December 2015, enabling a sufficient data set to be collected. It also enabled the journalistic constructions of GM food along with the below the line comments to be followed for a period of a year. The year 2015 was the second year of the research project and when the data were collected. This meant the data were collected ‘live’ to investigate the news reporting and comments during 2015. Using a ‘live’ approach meant there was no way of telling what was going to be reported during the specific time period. The sample gathered 78 articles and 9279 below the line comments.

Google Advanced Search was used to locate the news articles. The search was not meant to be exhaustive but was instead intended to return enough news articles and below the line comments for analysis. *GM food* and *genetically modified food* were both used as key word search terms. These were searched for using the ‘all these words’ option. The website address for each news organisation was put in the ‘site or domain’ option. The past year (from 1 January 2015) was selected for the ‘last update’ option. Once all this information was completed, the searches were conducted. However, searching for the news articles in this way meant each news article had to be individually checked. This was to ensure it fit within the sampling time frame. In total, 104 news articles were returned in the search. The news articles were also screened for relevance. 26 news articles which appeared in the search were not appropriate for this project. These were news articles concerning share prices in connection with companies such as Monsanto and Bayer. These related to business news as opposed to scientific developments. Therefore, these articles were omitted, and the total number of news articles relevant to the project was 78. In respect of the below the line comments, those used in the study were those which were associated with the news articles. Therefore, these did not have to be searched for separately. The number of below the line comments included in the sample was dependent on the number of audience members who decided to post a comment.

An important consideration which has to be taken into account in respect of this study, is that those who post comments may be those who are particularly interested in the subject of GM foods. In this respect, the views of those commenting are seen as being representative for this study and may not be characteristic of the population as a whole. Analysing the comments provides an approach for understanding the reception of the articles concerning GM foods by audience members. The data for reception analysis are often collected using methods such as interviewing, observation or focus groups. In contrast to these approaches, where participants have to be recruited and who often have to recall information, this study uses the actual responses of commenters. Therefore, these data are firsthand from the audience who are interested in commenting about GM foods. Their views, feelings, understandings, and beliefs are revealed in the comments they post.

### Data analysis

To examine the data in detail, a discourse analysis was employed drawing on theoretical concerns including science and technology studies, and sociology of science.

#### Discourse analysis

While there are many types of discourse analysis, the version used in this study is that developed by Gee (2011). By conducting discourse analysis, questions are effectively asked of the text being examined. According to Gee (2011), there are seven different building tasks used in the construction of language whenever we speak or write and for each, it is possible to ask a discourse analysis question. These seven building tasks are significance, practices (activities), identities; relationships; politics; connections, and sign systems and knowledge. The seven building tasks are fundamentally interlinked with each other (Gee, 2011).

In respect of selecting samples to analyse, a strategy Fairclough (1992) proposes is to focus on those elements of the discourse where there is an indication and evidence that something is amiss. He also suggests focusing on areas of discourse which are pivotal, indicate something which is vital, or are puzzling. These suggestions were followed and those extracts which best represented a pattern in the data were selected. The questions described above for Gee’s (2011) seven building tasks were then applied to the text extracts. An example of how the seven building tasks and the related discourse analysis questions were applied to the data are available in Price (2018).

In the findings which follow, any spelling mistakes or grammatical errors are left unchanged in the extracts taken from the news articles and below the line comments.

## 4. Findings and discussion

### News articles

The findings presented here are just one part of the overall study (see Price, 2018), and are representative only of the dispute between Professor Anne Glover and Greenpeace. It is acknowledged that readers of this article may have different interpretations of the data that are presented here. In addition, the data were collected and analysed in 2015 so the findings reflect this particular time period only.

**Table:** 

Greenpeace and other campaigning organisations are failing to be “honest” about genetically modified crops, the former chief science adviser to the European Commission has said. Professor Anne Glover accused the groups for “ignoring the evidence” and “fabricating a scenario” with their opposition to GM crops. She said that while she does not object to their “philosophy and ideology”, the campaigning groups should not try to support their views by “bad calling the evidence”. She told The Today programme on BBC Radio 4: “I’m deeply disappointed with them, because those NGOs that you mentioned were NGOs that I used to trust and many citizens do trust. I think they have ignored the evidence and they have fabricated a scenario. “If I look at their letter, and what they describe, because I’ve met with many of them they know that simply it’s not true what they talk about.

**Extract 1** From the article ‘Greenpeace is failing to be ‘honest’ about GM crops, former EU scientific adviser says’ (*The Telegraph*, 2015).

The discourse in this extract includes quotations from a radio interview conducted by Professor Glover, and she claims NGOs including Greenpeace have two specific strategies in relation to scientific evidence. One aspect is they appear to ignore it, while the other aspect is that they highlight failings with the evidence in order to support their own views. Professor Glover claims this is something she finds alarming due to how citizens trust the NGOs. She states she understands why citizens trust the NGOs because she used to herself. She also refers to a letter which was written by the NGOs and claims they are being dishonest because she has conducted meetings with them. This relates to a point put forward by Giddens (1991), whereby ‘experts can always be turned to, but experts themselves frequently disagree over both theories and practical diagnoses’ (p. 84). Greenpeace have scientific experts, and this illustrates the issue of the contested nature of science. Depending on which discipline scientists are from and the questions they ask, their results are all likely to differ. What this discourse illustrates, is that scientific research can be disputed not only from the work which is carried out, but also by whose work is included in the debate. The legitimacy of science presented by Professor Glover is reinforced by her previous position of authority of Chief Scientific Adviser. Here, a scientist is representing the environment through the use of scientific discourse.

News coverage of the dispute between Professor Anne Glover and Greenpeace also featured in *The Guardian*.

**Table:** 

Doug Parr, scientific adviser for Greenpeace UK, denied that “a disagreement about GM food” was at the heart of the dispute. “The problem lay in how this role lacked transparency, concentrated too much influence into the hands of just one person, making them vulnerable to industry lobbying, and allowed political interference in a process which should be driven by science.”

**Extract 2** From the article ‘Green groups lied to oust me, says former EC science chief’ (*The Guardian*, 2015).

The discourse in this extract is constructed to show how Doug Parr, the scientific adviser for Greenpeace, viewed the role of the EU Chief Scientific Adviser. Here, Doug Parr is representing Greenpeace. He claims the decisions made by the EU Chief Scientific Adviser should be determined by scientific evidence. The quotation illustrates Greenpeace’s claims that the role of the EU Chief Scientific Adviser was influenced by the political agenda. Greenpeace claim to be concerned about the power and influence of one person in the scientific policy decision-making process. Here, science should inform decisions, and politics should be excluded from decision making processes. This illustrates the importance of scientific evidence even to the NGOs. As Yearley (1991) argues, green activists ‘may find themselves rather ambivalent about science: they are often critical of it but find they need it too’ (p. 45). Here, the claims made by Greenpeace illustrate the importance of science to the organisation. Once again, scientific discourse is being used in environmental representation.

**Table:** 

A Greenpeace spokesman said that Greenpeace had actively sought to have meetings with the former chief scientist and had failed to attend one meeting because no senior representatives were available. “Professor Glover is being a bit selective in her recollection of events,” he said. Glover said she was unsure how much influence the lobbying from environmental groups had in the decision to abolish her role as she had not been given a detailed explanation by the commission.

**Extract 3** From the article ‘Green groups lied to oust me, says former EC science chief’ (*The Guardian*, 2015).

In this extract, a Greenpeace spokesperson claims attempts were made by Greenpeace to arrange meetings with Professor Glover. The quote from the Greenpeace spokesperson claims Professor Glover is selective in her articulation of her side of the story. A counterclaim is included in the extract which illustrates Professor Glover’s stance in connection with the incident. However, this is not a direct quotation made by Professor Glover and the claim is made by the journalist. Here, the journalist is providing balance to the news story, and in doing so, is representing Professor Glover. The journalist describes the abolishment of Professor Glover’s position as Chief Scientific Adviser and the campaigning by NGOs. The discourse also indicates the potential influence of NGOs on both policy decisions and job roles at a European level. At times, NGOs will exchange information and strategies with one another, in an attempt to guide policy in individual countries as well as at an international level (Fitting, 2014).

Although the preceding three extracts are from two alternative news organisations, they explain the contested nature of science and the authority which surrounds it. An argument put forward by Keck and Sikkink (1999) concerns the use of media organisations by NGOs. They contend NGOs require press attention in order to reach a broader audience with the message they wish to convey, and this is often achieved by using a powerful frame. For Scheufele and Tewksbury (2007), framing is ‘based on the assumption that how an issue is characterized in news reports can have an influence on how it is understood by audiences’ (p. 11). Journalists use framing to help reduce the complexity of an issue in order for the audience to understand the information presented to them. It can be argued that a dispute between the former EU Chief Scientific Adviser and Greenpeace is an effective frame to use in order to draw attention to an environmental issue. As these three extracts illustrate, scientific evidence is used by environmental representatives, but in very different approaches. Here, there are opposing views relating to the same incident. In the following extract, the ‘best’ science is used in environmental representation.

**Table:** 

“Greenpeace wants more and better scientific advice and evidence to be used by the Commission, which is why we have advocated strong, broadly-based, well-resourced, independent science advice with clarity about political judgements and clear processes. Ensuring that EU decision makers base their policy on the best available evidence in chemicals, pesticides and climate change is in the interests of all EU citizens and the environment.”

**Extract 4** From the article ‘Greenpeace is failing to be ‘honest’ about GM crops, former EU scientific adviser says’ (*The Telegraph*, 2015).

This extract contains a direct quotation from a spokesperson of Greenpeace. Their name is not disclosed. Greenpeace claim they want scientific evidence to be used in the decision making process concerning GM crops. This is constructed in terms of ‘more and better scientific advice and evidence’, ‘strong, broadly-based, well-resourced, independent science advice’, and ‘best available evidence’. Here, Greenpeace is selective in citing science that directly supports their claims.Science that is not aligned with their particular point of view is lesser as opposed to better, weak as opposed to strong, narrow as opposed to broad, under-resourced as opposed to well-resourced. The ‘best’ science is presented by Greenpeace as being neutral and objective. Here, the ‘best’ science is needed in order for Greenpeace to represent the environment effectively.

### Below the line comments

The below the line comments indicate a lack of trust in the use of scientific evidence by Greenpeace. In addition, the commenters’ claim Greenpeace appears to make judgements based on values as opposed to scientific facts. The commenters’ objectives through their claims-making appears to be to dismiss Greenpeace as a credible environmental organisation. The representative claims put forward by Greenpeace are interpreted and dismissed by the commenters.

**Table:** 

Mr Parr must accept that even when Greenpeace gets “more and better advice and evidence” they just ignore it if it doesn’t suit their purposes. The organisation is now a liability to serious environmentalists and is just a home for sad cases who dream of becoming a hero by lashing themselves to a chimney.

**Extract 5** Comment relating to the article ‘Greenpeace accused of making false GM claims’ (*The Times*, 2015).

This commenter refers to Mr Parr, Greenpeace’s UK Science Adviser, and his quote in the news article this comment relates to. The commenter claims that even if Greenpeace had ‘more and better advice and evidence’, the organisation will still dismiss it. The commenter claims Greenpeace will only use the scientific evidence they consider fits with the message the organisation wishes to convey. The discourse concerning campaigners scaling chimneys is also perhaps an indication of the media representation of Greenpeace in the past (see Hansen, 2000, 2010). Exposure to previous media coverage relates to an argument put forward by Hier (2003), in that ‘most people are aware of global affairs through their engagement with the mass media, but this form of mediation is encountered as a distinct mode of experience, separate from immediate experience and the contextuality of the familiar’ (p. 12). Here, the commenter appears to be drawing on the media messages they have experienced in the past. This illustrates the importance of environmental representatives creating effective frames and messages when appearing in the media. Frames and messages can be drawn upon by the audience when they articulate their own opinions in below the line comments.

**Table:** 

GM crops are about the only solution to solve the problem of starvation in many parts of the world, GM crops could be developed to withstand drought and many of the exotic plant diseases prevalent there, but do Greenpeace care about that? no, most of their members are simply “rent a mob” who have no understanding of life outside their tiny world.

**Extract 6** Comment relating to the article ‘Greenpeace accused of making false GM claims’ (*The Times*, 2015).

In this extract, the commenter claims GM crops are the only solution to addressing food security, and there is a need for developing plants resistant to drought or diseases. GM crops could be used to help feed those who are starving, but Greenpeace is preventing this from happening. The commenter claims members of Greenpeace have a restricted view of the world and suggests Greenpeace and its supporters do not care about food security issues. The commenter describes Greenpeace members as ‘rent a mob’ suggesting they are viewed by the commenter as people who are always protesting. The discourse of science is used by the commenter to dismiss the environmental representation put forward by Greenpeace.

What the comments in the previous two extracts have in common, is that those posting them claim Greenpeace are not using scientific evidence in their approach to contesting the introduction of GM crops. Their discourses appear to be constructed in respect of Greenpeace adopting a value based approach as opposed to one based on facts. However, according to Keck and Sikkink (1999), during the 1980s, Greenpeace changed focus from running media events to obtaining scientific evidence to ensure their facts were correct. Nevertheless, Greenpeace still uses the media. By arguing for the use of scientific evidence, Greenpeace gives the environment a voice through legitimate knowledge claims. However, the legitimacy of these claims by Greenpeace are contested by the commenters, who claim scientific evidence is not being used. The claims of commenters illustrates how knowledge can straddle the beliefs about what exists and what we believe should exist (Carolan, 2004). What exists and what we believe should exist is key to the contested nature of environmental representation in this study.

**Table:** 

Greenpeace began life as a citizens’ group devoted to fighting pollution and the whaling industry, but it’s now a powerful de-industrialisation lobby. Its hostility to progress snags it well over $200 m income a year. If a scientific breakthrough promises a better of quality of life, then the organisation is probably against it.

**Extract 7** Comment relating to the article ‘Green groups lied to oust me, says former EC science chief’ (*The Guardian*, 2015).

In this extract, the commenter claims that Greenpeace started off as a green group which had good intentions. It was designed as a campaign group which fought the whaling industry and pollution. The commenter claims the aim of Greenpeace has now changed, and instead it is against scientific progress. By opposing scientific developments, the commenter suggests Greenpeace is able to generate the income it requires in order to function as a business. The commenter goes on to claim that any scientific development which is beneficial to humans will be opposed by Greenpeace.

The comments about Greenpeace illustrate how commenters construct their arguments around a science perspective. They all claim Greenpeace do not base their decisions on scientific evidence. Irwin and Michael (2003) put forward the argument that citizens are not anti-science, and these comments illustrate this point. Citizens hold a wide range of views concerning GM foods (Gofton and Haimes, 1999; Shaw, 1999). In respect of trust in expert systems, these comments demonstrate how commenters believe in scientific experts, and trust that the scientific developments they propose should be proceeded with. For these commenters, science is the legitimate authority in determining what is acceptable for benefitting citizens’ lives. Collins (2014) makes a valid point in that if scientific legitimacy is undermined, it will be ‘those with the power to enforce their ideas or those with the most media appeal who will make our truths, according to whatever set of interests they are pursuing’ (p. 131). As the comments illustrate, there are those commenting who will continue to uphold scientific legitimacy. Here, commenters are using scientific arguments to challenge Greenpeace as a legitimate environmental representative.

## 5. Conclusion

This article explores Greenpeace’s use of science in claims-making and environmental representation in news articles concerning GM food, and how commenters respond in the associated below the line comments. The news articles concerning Greenpeace illustrate how this particular NGO bases their claims of authority on science, even if their position contradicts and competes with definitions from the EU Chief Scientific Adviser. Science is used by Greenpeace in their claims-making to consolidate the stance they take about GM foods and to legitimise the claims they are making. This illustrates that even when science is presented as facts only, it is subject to arguments and negotiation. As noted earlier, Yearley (1991) explains how NGOs may be ambivalent towards science, but they often find they require scientific evidence to support the claims they make. For the Greenpeace spokespeople, environmental representation is achieved through the presentation of science in claims-making. Science is presented as the ‘best’ type of knowledge, and appears to be used in order to build trust with the audience. With Professor Anne Glover, her legitimacy in the news article is reinforced by the journalist highlighting her previous position as Chief Scientific Adviser. Quotes by Glover state Greenpeace are ignoring scientific evidence, and these could potentially undermine the claims of Greenpeace.

Science is amplified in the news articles by Greenpeace and Professor Glover, but there is no agreement on what is appropriate scientific evidence. A weight of evidence narrative (Dunwoody and Kohl, 2017) is also apparent in the news articles, with the journalist putting forward scientific claims by Professor Glover and Greenpeace. Even though Greenpeace is an environmental representative, a discussion around the implications for the environment arising from the situation with Professor Glover is not evident in the news articles. As such, the environment remains voiceless.

Scientific evidence is used either in quotations by claims-makers in the news articles or by commenters. Science is often viewed as providing facts about the lines of enquiry being pursued. However, as Grove-White (1998) argues, science is not just fact based, it is shaped by and can be inspired by society. If it is only facts which are relied upon as opposed to also considering social values, scientific controversies can arise and this creates an uneasy relationship between science and society. This uneasy relationship is illustrated in the difference between the news articles and the below the line comments in connection with the scientific argument. The news articles suggest scientific evidence is important to Greenpeace who advocate for the use of science in decision making processes. This view is opposed in the below the line comments as commenters claim Greenpeace will dismiss scientific evidence. As Irwin and Michael (2003) argue, citizens

may not only possess knowledge, but have knowledge of how they know: they are able to reflect upon why they take on board some ‘scientific facts’ but not others; they are competent in accounting for why they prefer some sources of knowledge (e.g. personal experience) over others; and they can justify why they trust some expert authorities and are suspicious of others. (p. 28)

Although the below the line comments can be a form of quiet activism, challenging expert knowledge (Price, 2021), the comments analysed here show how commenters dismiss scientific evidence as proposed by Greenpeace but do not challenge expert knowledge per se. The commenters appear to try to define what they understand as scientific knowledge and evidence.

The claims that the commenters make undermine the legitimacy of Greenpeace as an environmental representative. Commenters’ claim that although Greenpeace used to represent the environment and nature, they no longer do this. The comments stand in opposition to the Greenpeace spokespeople who appear in the news articles, and therefore these comments offer the audience a competing interpretation of the role of Greenpeace. Greenpeace is an organisation that has existed for a long period of time. The rejection of the legitimacy and expertise of Greenpeace by the commenters appears in part to be based on the claims made by Greenpeace in the past. As the comments illustrate, the activism conducted by Greenpeace in the past appears to have undermined their legitimacy in making scientific claims. This raises the question of whether organisations such as Greenpeace can use science and scientific evidence in their claims-making in the media, and if this will be taken seriously. This is an area where further research is needed, especially in the area of climate change.

In order to establish the reasons or motivations as to why the audience comments, it would be necessary to conduct interviews with those who comment. As Graham and Wright (2015) contend, ‘people who comment are atypical and comment debates are not thus necessarily reflective of the broader readership’ (p. 330). As stated in the Methods section, the views of those commenting are seen as being representative for this study and may not be characteristic of the population as a whole. Also, the comments posted may not reflect the opinions of the audience as a whole. It is important to note that those who posted comments may be particularly interested in the subject of GM crops and food. Overall, it was difficult to ascertain information about those who were commenting, such as age, gender and occupation. The majority of those commenting used pseudonyms, enabling them to do so anonymously. This anonymity means that it is impossible to establish the motivations for commenting (Canter, 2013; Graham and Wright, 2015).

Further research is also needed to understand the structures of relations between news reporters and audiences compared to commenters and audiences. It would be necessary to conduct interviews with journalists, commenters, and audience members. This approach could also be used to ascertain if commenters are provoked by the reporting in news articles, or if the comments are informed by existing experiences and understandings. A further line of enquiry could be to interview actors such as Greenpeace to understand their expectations of appearing in news articles. It would then be possible to understand how this organisation views their role as an environmental representative in news articles. Greenpeace’s views and opinions on the role of commenters could also be ascertained.

This study shows that Greenpeace use scientific evidence to substantiate and legitimise their claims-making and environmental representation, and this is evident in the news articles. Commenters use scientific arguments to challenge Greenpeace as a legitimate environmental representative. While below the line comments enable a diversity of voices, in this study, these voices may be undermining arguments surrounding environmental threats.

## Notes

1. See Rehfeld (2006) for a full definition of the rules of recognition.
